# Condition‐dependent resource allocation strategy governed by CodY regulator in *Bacillus subtilis*

**DOI:** 10.1002/mlf2.70036

**Published:** 2025-10-22

**Authors:** Haoyan Mu, Yiheng Wang, Yongfu Pei, Xin Wang, Xiongfeng Dai, Manlu Zhu

**Affiliations:** ^1^ Key Laboratory of Pesticide & Chemical Biology of Ministry of Education Hubei Key Laboratory of Genetic Regulation and Integrative Biology, School of Life Sciences, Central China Normal University Wuhan China

**Keywords:** *Bacillus subtilis*, bacterial adaptation, CodY, growth control, resource allocation

## Abstract

To thrive in nature, bacteria have to rapidly proliferate in favorable conditions while constantly adapt to the fluctuating nutrient environments. However, the molecular players that ensure rapid growth of bacteria in favorable conditions remain poorly understood. Here, we focus on the growth physiology of *Bacillus subtilis* and find that *codY* knockout strongly compromises cell growth in rich medium. Global proteome allocation analysis has shown that *codY* knockout causes a “waste” of cellular resources by stimulating unnecessary expression of many proteins, further reducing the cellular investment on translation machinery. Therefore, CodY‐dependent repression is crucial in ensuring rapid growth of *B. subtilis* in rich medium. On the other hand, the relief of CodY‐dependent repression could promote the bacterial adaption during transition from rich medium to minimal medium by shifting resource allocation from ribosome synthesis to amino acid biosynthesis. In addition, the relief of CodY‐dependent repression in minimal medium also stimulates pathways of alternative functions such as motility and biosynthesis of secondary metabolites. Our study has thus revealed the pivotal role of CodY in bacterial growth control via governing the condition‐dependent resource allocation of *B. subtilis*, further shedding light on the fundamental molecular strategy of bacteria to achieve fitness maximization.

## Impact statement

Cell growth and environmental response are two fundamental capabilities of bacterial system. From a historical view, the fields of microbial physiology and molecular microbiology focus intensively on the stress response of bacteria when confronting environmental stressors. In contrast, we are still lack of understandings on how bacteria manage to achieve rapid growth in favorable conditions. Here, we show that the design of CodY regulatory network in *Bacillus* is crucial in both ensuring rapid growth in rich medium as well as in the bacterial adaption to nutrient downshift, thus providing mechanistic insights into the growth control strategy of Gram‐positive bacteria.

## INTRODUCTION

To maintain maximal fitness in nature, bacteria have to balance cell growth and stress response^1^, ^2^, ^3^, ^4^, ^5^. Exploring the signaling pathways of stress response in bacteria is a central goal in microbial physiology and molecular microbiology^6^, ^7^, ^8^. Although the nutrient statuses in the natural niches of bacteria are often highly fluctuating (“famine to feast cycle”)^4^, ^9^, ^10^, “rich medium” opportunities are not rare in the life cycles of model bacteria such as *Escherichia coli* and *Bacillus subtilis*, which could feed on the nutrients in the mammalian intestines^11^, ^12^ and the root exudates in the plant rhizosphere^13^, ^14^, respectively. Both *E. coli* and *B. subtilis* are capable of achieving a rapid growth rate of ~20–30 min per doubling in favorable conditions such as nutrient broth^12^, ^15^. Nevertheless, the global molecular strategy that ensures rapid growth of bacteria in rich medium remains poorly understood.

Recent phenomenological studies have revealed the fundamental role of ribosome synthesis in governing bacterial growth control^16^, ^17^, as exemplified by the linear relation between ribosome content and growth rate of bacteria under different nutrient conditions^18^, ^19^, ^20^. It has been found that the strategy of proteome resource allocation in *E. coli* favors maximization of ribosome synthesis in rich medium, as demanded by the high protein synthesis and rapid growth^17^, ^21^, ^22^, ^23^. Related quantitative frameworks of bacterial growth are of great values in guiding the rational designs of synthetic circuits^24^, ^25^. However, going beyond phenomenological modeling, the molecular strategy that ensures the optimal resource allocation of bacteria for supporting fast growth in rich medium remains poorly understood, especially for species other than *E. coli*.

In the Gram‐positive model bacterium *B. subtilis*, the CodY regulator is known to regulate the expression of dozens of genes by sensing the levels of branched‐chain amino acids (BCAA) and GTP^26^, ^27^, ^28^, ^29^. CodY, acting primarily as a transcription repressor, regulates various categories of genes such as amino acid (AA) biosynthesis, motility protein, nutrient transport and metabolism, sporulation, and some other stationary‐phase genes^26^, ^30^, ^31^. However, the role of CodY in growth control of *B. subtilis* remains elusive. In this study, we characterized the growth physiology of *B. subtilis* associated with the effect of CodY regulator. We found that CodY, via modulating proteome resource allocation, enables *B. subtilis* to achieve rapid growth in rich medium. Moreover, the relief of CodY‐dependent repression could enable bacteria to adapt to the growth transition from rich medium to minimal medium.

## RESULTS

### The effect of CodY on growth physiology of *B. subtilis*

**Figure 1:**
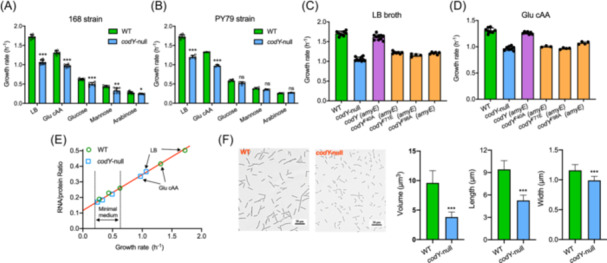
The effect of CodY on growth physiology of *Bacillus subtilis*. (A) The growth rates of wild‐type 168 strain and its *codY‐null* strain under various nutrient conditions, including two rich media (LB and glu cAA) and three minimal media with glucose, mannose, and arabinose as sole carbon source, respectively. (B) The growth rates of wild‐type PY79 strain and its *codY‐null* strain under various nutrient conditions, including two rich media (LB and glu cAA) and three minimal media with glucose, mannose, and arabinose as sole carbon source, respectively. (C, D) The effects of complementation of CodY and its three mutants defective in sensing BCAA on the growth rates of *B. subtilis* in both LB and glu cAA media. For the data of *codY* complementation (purple and three orange bars), the native *codY* and its three mutant genes were under the control of IPTG‐inducible P*grac* promoter and integrated into the *amyE* sites of *B. subtilis codY‐null* strain. 50 μM IPTG was supplied to induce the expression of CodY and its three mutants, including *codY* (*amyE*), *codY* ^F40A^ (*amyE*), *codY* ^F71E^(*amyE*), and *codY* ^F98A^ (*amyE*) conditions. (E) RNA/protein ratio of wild‐type 168 strain and its *codY‐null* strain under various nutrient conditions used in panel A. (F) The cell images and cell sizes of exponentially growing wildype 168 strain and its *codY‐null* strain in LB medium. Error bars (except panel F) denote the standard deviations of at least three biological replicates, while the error bar in panel F denotes the standard deviation of sizes of individual cells in a population (*n* = 466 for wild‐type strain and *n* = 483 for *codY*‐null strain). Significance analysis of panel A, panel B, and panel F is based on unpaired *t*‐test. Glu, glucose; glu cAA, glucose casamino acids. ns, no significance; *p < 0.05; **p < 0.01; ***p < 0.001.

We compared the growth rates of *B. subtilis* 168 strain and its *codY*‐null strain under various nutrient conditions. The exponential growth rates of wild‐type 168 strain were significantly higher than that of *codY*‐null strain in two rich media including LB broth and glucose casamino acids (glu cAA) medium (Figure 1A). In contrast, the differences between wild‐type strain and *codY*‐null strain in growth rates were much smaller in minimal medium (Figures 1A and S1). Similar results were also observed for the *B. subtilis* PY79 prototrophic strain (Figure 1B). We further confirmed that reintroduction of CodY could restore the growth defect of *codY*‐null strain (Figure 1C,D, purple). In contrast, reintroduction of inactive CodY variants that were defective in sensing BCAA^29^ failed to restore the growth defect (Figure 1C,D, orange). We then characterized the RNA/protein ratio, a proxy of ribosome content^20^, ^32^, of *B. subtilis* across nutrient conditions. The ribosome content of wild‐type and *codY*‐null strain followed the same linear relations with the growth rates (Figure 1E); however, wild‐type strain harbored a higher ribosome content than *codY*‐null strain in rich medium (arrows in Figure 1E), suggesting that the growth defect of *codY*‐null strain in rich medium originates from reduced protein synthesis capacity. The *codY*‐null strain also displayed a much smaller cell size than wild‐type strain, being consistent with its growth defect and the “growth law” of cell size (Figure 1F)^15^, ^33^, ^34^.

### Regulation of cellular resource allocation by CodY

**Figure 2:**
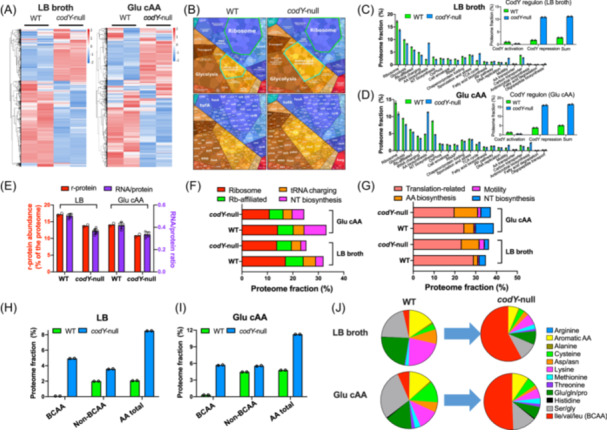
Regulation of proteome allocation by CodY in rich medium. (A) Heatmaps of the proteomes of wild‐type strain and *codY‐null* strain growing in LB broth and glu cAA medium. The two lanes represent the two biological replicates in each condition. (B) The proteome allocation of *B. subtilis* analyzed by proteomaps website. Note that the term of “mitochondrial biogenesis” was based on KEGG categorization consisting of both prokaryotes and eukaryotes. The readers should thus treat these proteins here as translation factors. (C, D) The mass fractions of various proteome sectors as well as proteins in CodY regulon (inset). Panels C and D correspond to the data of *B. subtilis* growing in LB broth and glu cAA, respectively. The gene list of CodY regulon was downloaded from Subtiwiki website. (E) r‐protein abundances and RNA/protein ratios of wild‐type and *codY*‐null strains growing in LB broth and glu cAA medium. Error bars denote the standard deviations of at least three biological replicates. (F) The mass fractions of transcription and translation machinery and nucleotide (NT) biosynthesis proteins. (G) The proteome fractions of transcription and translation machinery, NT biosynthesis, motility and AA biosynthetic sectors. (H, I) The proteome fractions of amino acid (AA) biosynthetic sector of *B. subtilis* in LB broth (H) and glu cAA medium (I), including both branched‐chain amino acids (BCAA) sector and non‐BCAA sector. (J) The composition of AA biosynthetic sector. PP, pentose phosphate.

To investigate the origin of reduced ribosome content of *codY*‐null strain, we next performed a proteome allocation analysis of *B. subtilis* using 4D label‐free quantitative mass spectrometry (MS) (Tables S1–S9, Figure S2). Heatmap analysis demonstrated that *codY* knockout substantially re‐shaped the global expression pattern of *B. subtilis* (Figure 2A). Visualization of cellular proteome by proteomaps website (https://proteomaps.net)^35^ demonstrated that the most notable change associated with *codY* knockout was the downregulation of ribosome content and upregulation of AA biosynthetic sector (Figure 2B). We further calculated the mass fractions of various functional sectors of the two strains in both LB medium and glu cAA medium (Figure 2C,D; Tables S3 and S7). As expected, the levels of proteins in CodY regulon were strongly upregulated in *codY*‐null background, while most genes in CodY regulon were subject to repression rather than activation (insets of Figure 2C,D; Tables S4 and S8)^26^, ^36^, ^37^. Being quantitatively consistent with the trend of RNA/protein ratio, the mass fraction of ribosomal protein (r‐protein), $\phi_{\mathrm{Rb}}$, became lower in the absence of *codY* (Figure 2E). Moreover, the trends of individual r‐proteins, including both 50S and 30S r‐proteins, were generally consistent with each other (Figure S3). *codY* knockout also reduced the levels of both translation affiliated proteins (e.g., FusA and TufA) and tRNA charging proteins, suggesting a global reduction in the cellular budget of translation machinery (Figure 2C,D,F,G). In addition, *codY* deletion caused a reduction in nucleotide (NT) biosynthetic pathway (Figure 2C,D,F,G), especially in glucose cAA medium, which is logical considering the lower demand for rRNA synthesis during slower growth^38^. Overall, *codY* knockout strongly reduces the cellular budget of translation machinery, which naturally explains its lower growth rate than wild‐type cells.

Quantitatively, the decrease in the mass fraction of translation machinery and NT biosynthetic sector together accounts for 7%–8% of the proteome of *B. subtilis* (Figure 2F,G, Tables S3 and S7). Such a substantial change is intriguing as none of these genes are previously known to belong to CodY regulon. Then what could account for the substantial changes in the levels of these biosynthetic machineries? We recalled that previous studies had found that proteome constraint by overexpressing useless proteins in bacteria could substantially limit the proteome budget of ribosomes and translation‐affiliated proteins, further compromising bacterial growth^32^, ^39^. We thus asked whether *codY* knockout could stimulate the expression of some “useless” proteins. Among those proteome sectors that are upregulated by *codY* knockout, the increase of mass fraction of AA biosynthetic pathway, $\phi_{\mathrm{AA}}$, accounts for 6.5% of the proteome in both LB and glu cAA medium (Figure 2G, Tables S3 and S7). However, the trend of each component in AA biosynthetic pathways was not uniform: the change of BCAA biosynthetic pathway (*ilv‐leu* related genes) is most substantial while the change of non‐BCAA sector is much weaker (Figures 2H,I, S4, and S5). This result is consistent with the fact that BCAA biosynthesis is a major target of CodY‐dependent repression^26^, ^27^. In addition to BCAA biosynthetic pathways, the expression levels of genes involved in biosynthesis of methionine (e.g., *hom*, *metC*, *metE*, and *metI*), threonine (*thrB*, *thrC*, and *thrD*), and histidine (*hisA*, *hisD*, *hisG*, *hisH*, and *hisI)* were also upregulated, though at a lesser extent compared to BCAA biosynthesis (Figures S4 and S5). Overall, the stimulation of *codY* knockout on AA biosynthesis was in a non‐coordinated manner (Figure 2J). Besides AA biosynthetic pathways, the mass fraction of motility proteins ($\phi_{\mathrm{mot}}$, including chemotaxis and flagellar proteins), another category of proteins belonging to CodY regulon, also increased strongly (from 0.76% to 2.5% and from 0.7% to 1.5% in LB and glu cAA media, respectively) (Figures 2C,D,G and S6A)^40^, ^41^, ^42^, ^43^. Besides AA biosynthetic and motility proteins, we also observed upregulated expression of some other proteins in CodY regulon, being involved in various functions such as secondary metabolite biosynthesis (e.g., bacB, bacC, and bacD, bacF, bacG) and alternative nutrient utilization (e.g., frlB, hutP, hutU, putC, and rocA) (Figure S6). Overall, the increases in $\phi_{\mathrm{AA}}$ and $\phi_{\mathrm{mot}}$ in *codY*‐null strain together, accounting for 7%–8% of the proteome, were comparable to the decreases in the abundances of translation machinery and NT biosynthetic sector (Figure 2G). Since AA biosynthesis and motility proteins are not necessary for supporting cell growth in rich medium where nutrient resources are highly abundant, *codY* deletion indeed causes a “waste” of proteome resources by stimulating unnecessarily high expression of many related proteins. Such a type of proteome “waste” limits the cellular budget of translation machinery and thus compromise cell growth of *codY*‐null strain.

### Nutrient‐dependent regulatory effect of CodY

**Figure 3:**
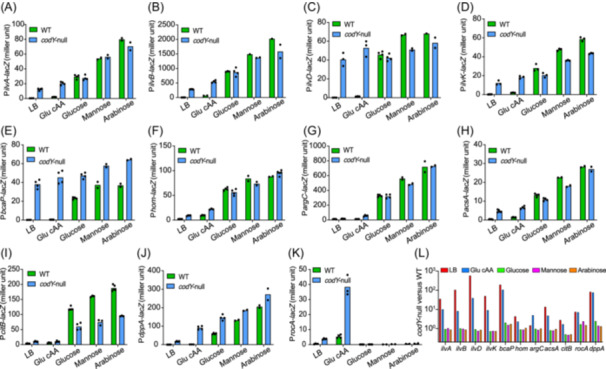
Nutrient‐dependent effect of CodY on gene expression. LacZ reporter assay was used to analyze the promoter activities of target genes under various nutrients, including two rich media (LB and glu cAA) and three minimal media with glucose, mannose, and arabinose as sole carbon source, respectively. (A) P*ilvA‐lacZ* activity. (B) P*ilvB‐lacZ* activity. (C) P*ilvD‐lacZ* activity. (D) P*ilvK‐lacZ* activity. (E) P*bcaP‐lacZ* activity. (F) P*hom‐lacZ* activity. (G) P*argC‐lacZ* activity. (H) P*acsA‐lacZ* activity. (I) P*citB‐lacZ* activity. (J) P*dppA‐lacZ* activity. (K) P*rocA‐lacZ* activity. (L) The ratios of promoter activities of various genes of *codY‐null* strain to those of wild‐type strain in panels A to K.

We next investigated why the growth defect of *codY*‐null strain largely disappeared in minimal medium (Figure 1A,B). It is known that the activity of CodY is medium‐dependent as its activity is stimulated by its association with GTP and BCAAs, which are limited in minimal media^28^, ^29^, ^31^, ^36^, ^44^. Therefore, it is conceivable that genes belonging to CodY regulon (e.g., BCAA biosynthesis) might be differentially expressed in rich and minimal media. We thus compared the promoter activities of a series of CodY‐repressed genes in various nutrient conditions (Figure 3). Among them, *ilvA, ilvB, ilvD, ilvK* are involved in BCAA biosynthesis; *bcaP* is related to BCAA uptake; *hom* and *argC* are related to the biosynthesis of methionine, threonine, and arginine; *acsA* and *citB* are related to TCA cycle; *dppA* and *rocA* are related to the utilization of arginine/ornithine and oligopeptide, respectively. We found that *codY* deletion strongly stimulated the promoter activities of these genes in LB broth and glu cAA medium (Figure 3A–K). The promoter activities of these genes (e.g., BCAA biosynthetic genes) in wild‐type cells were also induced in minimal medium (Figure 3, green bars) where AA biosynthesis is indispensable for supporting cell growth. We note that the promoter activities of these CodY‐regulated genes become much more comparable between wild‐ type strain and *codY*‐null strain in minimal medium than in rich medium (Figure 3A–K). Therefore, the ratio of the promoter activities of *codY*‐null background to wild‐type background is much lower in minimal medium than that in rich medium (Figure 3L). This result suggested that the resource “waste” in *codY*‐null strain compared with wild‐type strain largely disappears in minimal medium, further accounting for the much weaker effect of *codY* knockout on cell growth in poor nutrients. Overall, we concluded that the CodY‐dependent resource allocation of *B. subtilis* is condition‐dependent and mainly affects cell growth in rich medium while such effect largely disappears in minimal medium due to the relief of CodY‐dependent repression.

### Relief of CodY‐dependent repression favors growth transition from rich medium to minimal medium

**Figure 4:**
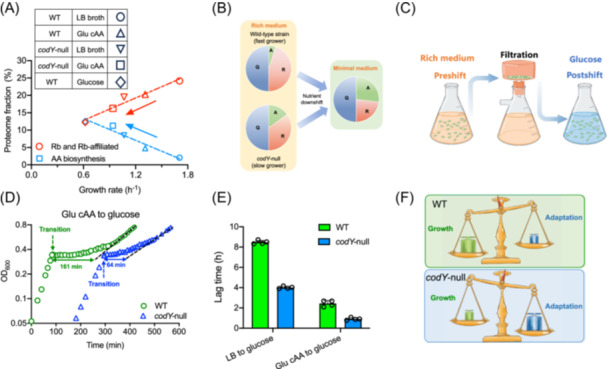
Absence of CodY promotes the adaption of *B. subtilis* to nutrient transition from rich medium to minimal medium. (A) The proteome fractions of ribosome (Rb) and Rb‐affiliated proteins and amino acid (AA) biosynthetic sector of wild‐type strain and *codY*‐null strain under different conditions. (B) Illustration of the cellular resource allocation of *B. subtilis* when shifted from rich medium to minimal medium. The coarse‐grained model of proteome partitioning in *B. subtilis* consists of a constant housekeeping Q sector, the growth‐dependent ribosome (R) sector and AA biosynthetic (A) sector. When shifted to minimal medium, bacteria had to upregulate A sector at the cost of reduced R‐sector. Compared with the fast‐growing wild‐type strain, *codY*‐null strain had a lower level of R‐sector but a higher level of A‐sector in LB medium. (C) Illustration of the nutrient downshift experiment. Cultures were first grown in rich medium (either LB broth or glu cAA medium), then subjected to vacuum filtration and collected into a filter membrane. Cells were then washed twice by minimal medium and transferred to the final glucose minimal medium. (D) Growth of *B. subtilis* during transition from glu cAA‐rich medium to glucose minimal medium. (E) Lag time of wild‐type and *codY*‐null strains during transition from rich medium (LB broth or glu cAA medium) to glucose minimal medium. Error bars denote the standard deviations of four biological replicates. (F) Schematic illustration of the trade‐off between growth and adaptability to nutrient downshift for *B. subtilis*. Wild‐type strain pursues growth maximization in rich medium while *codY*‐null strain has reduced growth rate but enhanced adaptability to nutrient downshift.

Another important aspect of the life cycle of bacteria is adapting to the fluctuating nutrient environments^4^, ^9^, ^10^. Although genetic mis‐regulation associated *codY* deletion is unfavorable for biomass growth in rich medium, it might help bacteria better adapt to minimal medium where the CodY‐dependent repression is relieved. A pre‐requisite for cell to maintain growth in minimal medium is the de novo biosynthesis of AA biosynthetic proteins to fullfill the metabolic flux of cells. As shown in Figures 4A and S7, *codY* deletion triggers the proteome reallocation of *B. subtilis* from ribosome synthesis to AA biosynthesis in rich medium, being closer to the resource allocation strategy of *B. subtilis* in minimal medium. In such case, the relief of CodY‐dependent repression by *codY* deletion might promote the bacterial adaption to nutrient‐limited conditions where AA biosynthesis becomes essential for supporting biomass growth (Figure 4B)^4^, ^12^. To test this scenario, we next performed a nutrient downshift experiment (see method), in which the exponentially growing *B. subtilis* cultures in rich medium were transferred to glucose minimal medium (Figure 4C). The *codY*‐null strain indeed required much shorter lags than wild‐type strain during nutrient transition from rich medium to minimal medium (Figure 4D,E).

Collectively, this result demonstrates that *codY* deletion promotes bacterial adaption to nutrient downshift at the cost of reduced growth rate, resulting in a trade‐off between growth and adaptability for *B. subtilis* (Figure 4F). In another word, wild‐type strain manages to pursue growth maximization in rich medium even at the cost of reduced adaptability to nutrient fluctuations.

### The derepression of CodY regulon supports both cell growth and other physiological traits in minimal medium

**Figure 5:**
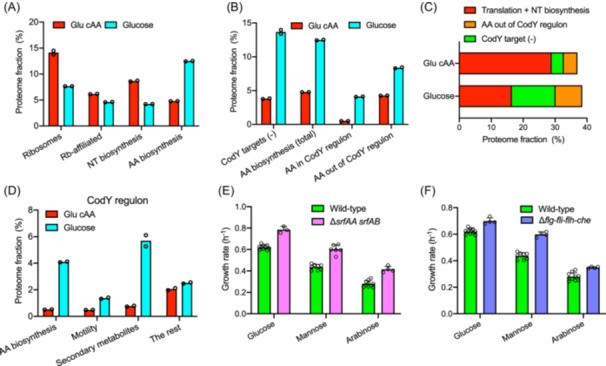
The fundamental role of CodY in the condition‐dependent resource allocation strategy of *B. subtilis*. (A) The proteome fractions of r‐protein, Rb‐affiliated proteins, NT biosynthetic proteins, and AA biosynthetic proteins in wild‐type strain growing in glu cAA‐rich medium or glucose minimal medium. (B) The proteome fractions of four sectors of wild‐type strain in glu cAA rich medium and glucose minimal medium, including the total AA biosynthetic sector, AA biosynthetic sector belonging to CodY regulon, AA biosynthetic sector out of CodY‐regulon as well as the target proteins of CodY regulon (repression). See Table S9. (C) The proteome fractions of translation plus NT biosynthetic sector, AA biosynthetic sector out of CodY regulon as well as the target proteins in CodY regulon (repression) in wild‐type strain. (D) The proteome fractions of various types of proteins in CodY regulon (reppression) in wild‐type strain. (E, F) The growth rates of wild‐type strain, *srfAA‐srfAB*‐null strain and *flg‐fli‐flh‐che‐*null strain on various carbon sources. Data of Δ*srfAA‐AB* strain have been reported in Ref.^5^. Error bars denote the standard deviations of at least three biological replicates.

The above result demonstrates that CodY‐dependent resource allocation is crucial to ensure rapid growth of *B. subtills* in rich medium. However, the effect of CodY on cell growth in minimal medium is much weaker due to the relief of CodY‐dependent repression, and therefore, we next investigated the physiological role of derepression of CodY regulon in minimal medium. We compared the proteomes of wild‐type strain in glu cAA rich medium and glucose minimal medium. The abundances of translation machinery and NT biosynthetic sector in minimal medium dropped substantially compared to that in rich medium (Figure 5A, Table S9), being consistent with the lower growth rate. In contrast, the AA biosynthetic sector increased strongly as it is indispensable to support bacterial growth in AA‐free minimal medium (Figure 5A). However, the increase in the proteome fraction of AA biosynthetic sector, $\phi_{\mathrm{AA}}$ = 6.5%, is far from enough to account for the drop in the proteome fraction of translation machinery and NT biosynthesis (12.5%) (Figure 5A, Table S9), suggesting a substantial increase in other categories of proteins. Strikingly, we found that the proteome fraction of genes in the CodY regulon (repressive genes) increased substantially from 3.8% to 13.6% (Figure 5B, Table S9). The increase in proteome fraction of CodY regulon together with AA biosynthetic sector, ϕ = 12.8%, is just enough to account for the decrease of translation machinery and NT biosynthesis (Figure 5C), suggesting a major contribution of derepression of CodY regulon in the resource allocation strategy of *B. subtilis* during growth in minimal medium. Among the upregulated genes of CodY regulon, AA biosynthetic sector, being undoubtedly important for supporting cell growth in minimal medium, only accounts for ∽36% (Figure 5D and Table S9). Other upregulated genes in CodY regulon are involved in alternative objects such as the biosynthesis of secondary metabolites (e.g., surfactin) and motility (Figure 5D). We note that biosynthesis of secondary metabolites and motility even have negative effects on the growth rates in minimal medium (Figure 5E,F), likely being related to the additional cost associated with these genes (*srfAA‐AB* and *flg‐fli‐flh‐che*, respectively). These results suggest that the resource allocation strategy of *B. subtilis* in minimal medium not only supports cell growth but also favors bacterial adaptability to other conditions, for example, increasing motility to prepare for extreme nutrient limitation^45^ and synthesizing secondary metabolites to combat environmental competitors^46^.

## DISCUSSION

**Figure 6:**
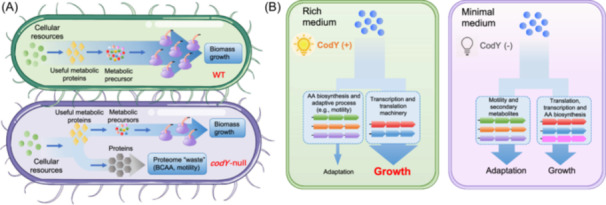
CodY is a global growth regulator of *B. subtilis.* (A) CodY regulates cellular resource allocation of *B. subtilis* in rich medium. The absence of CodY repression causes a “waste” of cellular resources by stimulating the overexpression of related proteins such as AA biosynthetic proteins, further diminishing the cellular investment on transcription and translation machinery and causing growth reduction. Therefore, CodY‐dependent repression is crucial in ensuring rapid growth of *B. subtilis* in rich medium. (B) CodY governs the condition‐dependent resource allocation strategy of *B. subtilis*. In rich medium, CodY‐dependent repression minimizes the proteome burden of AA biosynthesis and some adaptive pathways (e.g., motility) and further enables *B. subtilis* to achieve rapid growth. In minimal medium where the nutrient condition is aggravated, the derepression of CodY regulon not only serves to support cell growth in minimal medium by activating AA biosynthesis but also stimulates alternative pathways such as motility and biosynthesis of secondary metabolites. In this case, the resource allocation strategy of *B. subtilis* could not only support biomass growth but also help the bacterial cells to prepare for environmental aggravation.

Bacteria have to adapt to the highly fluctuating environments (“feast and famine” cycle) in their nature niches. At high nutrient availability (rich medium), bacteria manage to maximize ribosome biosynthesis to achieve rapid growth to enlarge their populations while AA biosynthetic proteins and adaptive proteins are maintained in basal levels^12^, ^47^, ^48^. In this study, we identified CodY network as an important molecular strategy that ensures the rapid growth of *B. subtilis* in rich medium. Although the regulatory mechanism and cellular targets of CodY have been identified before^26^, ^37^, ^44^, the important role of CodY in growth control of *B. subtilis* has not been elucidated. As a conserved regulator, CodY protein is widely distributed in low‐GC Gram‐positive bacteria^26^, ^37^, ^44^. The activity of CodY relies on the presence of BCAAs and GTP, and therefore, is tightly coupled with the metabolic status of bacterial cells^28^, ^29^, ^36^, ^44^. As a DNA‐binding repressor, CodY contains a GAF domain that senses BCAAs and a winged helix‐turn‐helix (wHTH) domain for DNA binding (consensus motif: AATTTTCWGAAAATT)^30^, ^44^, ^49^. Over 100 genes have been identified to be repressed by CodY, including AA biosynthetic genes (especially BCAAs biosynthesis), motility proteins and many early‐stationary‐phase genes^26^, ^30^, ^37^, ^42^, ^43^, ^50^. Here, we find that the transcription regulation of CodY could strongly modulate the proteome allocation of *B. subtilis* growing in rich medium. In the absence of CodY repression, the expression of AA biosynthetic proteins (especially BCAAs biosynthesis) and motility proteins substantially increase by an approximately 8% of the whole proteome (Figure 2G–I). Since AA biosynthetic proteins and motility proteins are non‐essential for supporting cell growth in rich medium (motility proteins are not even required in minimal medium, see Figure 5F)^12^, ^45^, ^51^, the overexpression of them in *codY*‐null strain is useless for cell growth and imposes a strong proteome burden. Previous quantitative studies have proposed that overexpression of useless protein compresses the proteome resources (e.g., ribosomes) that support biomass growth, leading to the slow‐down of growth rate^32^, ^47^, ^52^, ^53^. Being consistent with this notion, we do see that the increased proteome burden in *codY*‐null strain is accompanied with substantially reduced level of ribosomes and translation affiliated proteins (Figure 2F,G). Moreover, we observed that the R‐line (linear growth‐R/P relationship) of the *codY*‐null strain is quantitatively similar to that of the wild‐type strain (Figure 1E). The major difference lies in the ribosome content of the *codY*‐null strain, which dropped alongside the R‐line compared to the wild‐type strain in two rich media. This observation also aligns with the established phenomenon in *E. coli* wherein overexpression of unnecessary proteins triggers a concomitant reduction in cellular ribosome levels, which scale linearly with growth rate across varying degrees of protein overexpression and is quantitatively similar to the R‐line of *E. coli* wild‐type strain during nutrient limitation^32^, ^54^. Collectively, the growth control effect of CodY is consistent with the passive regulation model of proteome resource allocation as CodY prevents the expression of many unnecessary proteins (e.g., AA biosynthesis) in rich medium, which could otherwise impose a strong proteome burden on *B. subtilis* in the absence of CodY^32^, ^47^, ^52^. The absence of CodY leads to a “waste” of proteome resource that compromises ribosome synthesis and cell growth in rich medium (Figure 6A). Moreover, the “waste” of proteome resource largely diminishes in minimal medium due to relief of CodY‐dependent repression, which relies on the presence of BCAAs and GTP^28^, ^36^, and thus the effect of *codY* deletion on cell growth is condition‐dependent and mainly takes effect in rich medium.

Although our work supports that the negative effect of CodY knockout on cell growth in *B. subtilis* is via passive regulation of resource allocation due to increased proteome burden, we do not exclude other possibilities that might also contribute to the slow‐down of cell growth. For example, it is possible that CodY knockout could also inhibit ribosome synthesis in a direct way. However, since CodY acts primarily as a transcription repressor^26^, ^37^, it is unlikely that it could directly activate the ribosome synthesis of *B. subtilis*. Given that GTP is the primary factor that regulates rRNA synthesis in *B. subtilis* ^38^, ^55^, there could exist some unknown mechanisms that CodY knockout causes the reduction of nucleotide biosynthesis. In support of this scenario, nucleotide biosynthesis is also downregulated in the absence of CodY. Nevertheless, it is possible that the reduction of nucleotide biosynthesis might merely be a manifestation of lower cellular demand for rRNA biosynthesis resulting from increased proteome burden associated with CodY knockout. From a broader viewer, our study has shown that cellular targets of CodY in *B. subtilis* is much broader than previously thought, including global cellular machineries such as ribosomes and translation‐affiliated proteins, which does not belong to CodY regulon (Table S4). Our finding actually supports that the effect of CodY on global gene expression could be via both active regulation (via transcription repression) and passive regulation (via modulating proteome resource allocation). In the context of passive regulation, the absence of CodY repression imposes a strong proteome burden that consumes a substantial fraction of ribosomes. Consequently, fewer ribosomes are available for self‐translation, leading to a global downregulation of ribosome synthesis and translation‐related proteins. Such a type of passive regulation has previously been demonstrated for the ppGpp signaling in *E. coli* ^55^, ^56^.

Interestingly, we observed that for certain proteins within the CodY regulon, such as those involved in oligopeptide transport (OppA, OppB, OppC, OppD, OppF) and surfactin biosynthesis (SrfAA‐AB‐AC‐AD)^42^, ^57^, ^58^, their levels were even differentially affected by CodY knockout in LB versus glu cAA medium (Figure S6). This suggests that the mode of CodY‐mediated regulation on these genes is likely to be influenced by the distinct nutrient compositions of media. The nutrient components, for example, concentrations of BCAAs, in LB and glucose cAA media could be different, which could further differentially affect the activity of CodY.^29^, ^36^ Furthermore, the promoters of different CodY‐regulated genes have varied sensitivities to the level or activity of CodY protein due to the different locations and strengths of binding motifs^30^, ^49^. In such sense, different categories of CodY‐regulated pathways could have direct competitions for the limited numbers of cellular CodY, further affecting the relative abundances of each other under different nutrient conditions.

Our study has also uncovered the molecular basis for distinct resource allocation strategy of *B. subtilis* under different growth conditions, as illustrated in Figure 6B. In rich medium, *B. subtilis* employs CodY network to repress the expression of those unnecessary genes to ensure high rates of ribosome synthesis and biomass growth. From a global perspective, our results suggest that cells attempt to pursue growth maximization in favorable conditions even at the cost of compromising the adaptability to nutrient fluctuations (Figure 4). However, when the environments become deteriorated (minimal medium), cells attempt to support both growth and adaptability. On one hand, the relief of CodY repression allows activation of AA biosynthesis to sustain cell growth in minimal medium; on the other hand, the derepression of CodY regulon^5^, ^59^, ^60^, stimulates the expression of many proteins that are not related to biomass growth, for example, motility and biosynthesis of secondary metabolites. Therefore, the resource allocation strategy of *B. subtilis* in minimal medium no longer merely pursues fast growth, and moreover, also supports adaptability to be better prepared for the upcoming environmental deteriorations. In this sense, CodY plays a pivotal role in governing the condition‐dependent resource allocation strategy of *B. subtilis*.

Finally, we highlight here that although our study focuses on the specific effect of CodY on growth control of *B. subtilis*, similar mechanisms could be applicable to a broad range of other species. As a global repressor, CodY protein is highly conserved in low‐G + C Gram‐positive firmicutes, including *Bacillus*, *Clostridium*, *Staphylococcus*, *Streptococcus*, *Lactococcus*, and *Listeria* ^26^, ^37^, ^44^, ^61^. In these species other than *Bacillus*, studies have shown that CodY also participates into regulating the expression of AA biosynthetic genes, early‐stationary phase genes and even some virulence genes that are related to adaptive response^26^, ^37^. As long as members of CodY regulon could account for a large fraction of the proteome, CodY could play a similar role in regulating cell growth in other related Gram‐positive species. On the other hand, CodY homologs do not exist in Gram‐negative bacteria, for which, other mechanisms should exist in preventing unnecessary expression of AA biosynthetic operons when growing in rich media. One strategy could be transcription attenuation (e.g., in *trp* operon in *E. coli*), which could effectively prevents unnecessary expression of anabolic proteins in bacteria growing in rich medium^62^. Another strategy is related to the ppGpp signaling, which could positively regulate AA biosynthesis^4^, ^55^. It has been recently shown that ppGpp signaling in *E. coli* is tightly coupled with the translation elongation rate of ribosomes^20^. A high translation speed for cells growing in rich medium results in a very low basal level of ppGpp so that the AA biosynthetic pathways of *E. coli* could be maintained at a basal level in rich medium^4^, ^12^.

## MATERIALS AND METHODS

### Strain construction

All strains are derivatives of *B. subtilis* 168 (*trpC*2)^63^, ^64^ and its *codY*‐null strains. The deletion of *codY* allele was based on the double‐crossover homologous recombination using the pDG1730‐derived pDG1730‐*Sal*I (*spc* ^*R*^) integration vector^5^, ^65^. To generate the *codY*‐null strain, the flanking regions of both upstream and downstream of *codY* were PCR amplified using the genome of strain 168 as a template, being further inserted into the *Aat*II/*Bam*HI sites and *Eco*RV/*Sal*I sites of pDG1731‐*Sal*I (*spc* ^*R*^) to replace the original two fragments of *amyE*. The resultant vector was linearized by *Xho*I digestion and then transformed into 168 strain and PY79 strain via natural competence^30^ and screened for spectinomycin‐resistant double‐crossover Δ*codY* recombinants with PCR verification. The deletion of the *flg‐fli‐flh‐che* motility region (started from *flgB* and ended at *cheD*) was performed similarly as *codY* deletion. Electrophoresis reagents such as 50× tris‐acetate‐EDTA buffer, agarose, and GoldBand DL5000 DNA Marker were ordered from Yeasen Biotech (Shanghai).

To construct a *codY* complementary strain, the *lacI*‐P*grac* cassette in pHT01 was amplified and assembled with the coding region of *codY* gene and further inserted into the *Bam*HI/*Hind*III sites of pDG1733‐CmR integration vector (the *spc* ^*R*^ was replaced by the *Cm* ^*R*^ of pHT01) using Gibbson assembly with the 2× MultiF Seamless Assembly Mix (RK21020) (ABclonal), generating the pDG1733‐P*grac*‐*codY* plasmid. The F40A, F71E, and F98A mutations were introduced into the *codY* coding region by PCR‐mediated site mutagenesis approach associated with *Dpn*I digestion^66^, generating additional three plasmids associated with *codY* mutations. The resultant four vectors were linearized by *Xho*I digestion and then transformed into *B. subtilis* 168 Δ*codY* strain and screened for chloramphenicol‐resistant double‐crossover strain. For the correct transformants, it had been verified that the *lacI‐*P*grac‐codY* cassette had been integrated at the *amyE* locus by double‐crossover recombination.

To construct various *lacZ* reporter strains, an improved version of pAX01‐derived integration vector of *B. subtilis*, pZDlacA, was constructed. The P*ilvA* promoter region (the intergenic region between *ilvA* ORF and its upstream gene) was PCR amplified using the genome of strain 168 as the template and further inserted into the *Xho*I/*Spe*I sites of a pAX01‐derived, pAX01‐*lacZ* vector^5^, ^67^. Two 1 kb fragments of homologous arms of *lacA* gene, *lacA1* and *lacA2*, were also PCR amplified using the genome of strain 168 as the template. Then the P*ilvA‐lacZ*‐*erm* ^R^ cassette, *lacA1*, *lacA2*, and the rest part of pAX01‐*lacZ* vector containing replicons and *amp* ^R^ were assembled together using Gibbson assembly kit, 2× MultiF Seamless Assembly Mix (RK21020) (ABclonal), generating the pZDlacA vector. In brief, the pZDlacA vector contained the P*ilvA‐lacZ* cassette and also two longer homologous arms of *lacA* gene than the original pAX01 vector so that its integration efficiency was higher than pAX01. The pZDlacA plasmid was then linearized with *Bsa*I and transformed into the both wild‐type strain and Δ*codY* strain using natural competence and screened for erythromycin‐resistant recombinants with P*ilvA‐lacZ* cassette being integrated into the *lacA* locus. For constructing the *lacZ* reporter strains of other genes, the promoter regions of selected gene candidates were also PCR amplified and inserted into the *Xho*I/*Spe*I sites of pZDlacA to replace the *PilvA* promoter. The resultant vectors were further transformed into *B. subtilis* to obtain the corresponding *lacZ* reporter strains.

### Medium

*B. subtilis* strains were grown in LB rich medium and modified C‐minimal medium^5^. LB rich medium, purchased from Coolaber Biotech, Beijing, contained 10 g/l tryptone, 5 g/l yeast extract, and 10 g/l NaCl. The basic recipe of C‐minimal medium contained 16 g/l K_2_HPO_4_, 4 g/l KH_2_PO_4_, 2.32 mg/l MnSO_4_·4H_2_O, 0.123 g/l MgSO_4_·7H_2_O, 12.5 μM ZnCl_2_, 50 g/l tryptophan, and 22 mg/l ferric ammonium citrate. In addition, the medium contains 20 mM NH_4_Cl and various carbon sources such as 0.4% glucose, 0.4% mannose, and 0.4% arabinose. The glu cAA medium contains 0.4% cAA in addition to glucose and NH_4_Cl. Antibiotics were used at the following concentrations: 100 μg/ml spectinomycin, 2 μg/ml erythromycin, and 5 μg/ml chloramphenicol (Coolaber Biotech, Beijing).

### Cell growth

Cell growth was performed in an air bath shaker (200 rpm, 37°C). A standard cell growth procedure includes three steps: seed culture, pre‐culture, and experimental culture (final culture). For the seed culture, cells from a fresh colony were inoculated into LB liquid medium, and grew at 37°C until OD_600_~1. Cells were then cultured in C‐minimal medium as the pre‐culture. Next day, bacterial pre‐culture was transferred to another fresh C‐minimal medium (initial OD_600_ at 0.02) for the following experiments. For cell growth in LB medium, the pre‐culture step was omitted. During cell growth, five to eight OD_600_ data points (usually within the range of 0.05–0.5) were measured by Genesys 50 spectrophotometer (Thermo Fisher Scientific) to obtain the exponential growth curve. The growth rate was calculated based on the exponential range.

### Nutrient downshift experiment

The procedure of nutrient downshift experiment has been described previously^4^, ^12^. 15–20 ml of bacterial culture was first grown exponentially in rich medium (LB broth or glu cAA medium) to OD_600_~0.3. The culture was then subject to vacuum filtration (TW‐606N, Toone Bio, Zhou Wen) and quickly collected by a 0.22 µm filter membrane. The cells in the filter membrane were further washed twice by the pre‐warmed post‐shift minimal medium (5 ml each time). The cells in the filter membrane was then pipetted into a small petri dish by 5 ml pre‐warmed post‐shift minimal medium and further inoculated into the post‐shift minimal medium (as time zero (*T*_0_)). The liquid culture was then quickly transformed to a 24‐well microplate for OD_600_ measurement using a Synergy H1 microplate reader (Agilent Biotek).

### Total RNA measurement

The procedure of total RNA quantification has been described^68^. Briefly, 1.5 ml of cell culture (OD_600_ of 0.3–0.4) was collected by centrifugation (12000 rpm, 2 min). The pellet was washed twice with 0.6 mL cold 0.7 M HClO_4_ and then digested with 0.3 ml 0.3 M KOH for 1 h at 37°C with constant shaking (100 rpm). Subsequently, the cell extract was neutralized with 0.1 ml 3 M HClO_4_ and centrifuged at 13,000 rpm for 5 min. The supernatant (0.4 ml) was transferred into a new 2 ml centrifuge tube. The precipitates were further washed twice with 0.55 ml cold 0.5 M HClO_4_ and again transferred into the same centrifuge tube. A final volume of 1.5 ml of supernatant was then measured for OD_260_. The total RNA amount (μg/ml/OD_600_) equaled to OD_260_ × 31/OD_600_.

### Total protein measurement

Biuret method was used for total protein quantification as described^68^. Briefly, 1.8 ml of cell culture (OD_600_ of 0.3–0.4) was collected by centrifugation (12,000 rpm, 2 min). The pellet was washed twice with 1 ml 0.15 M NaCl. Then the precipitate was suspended with 0.2 ml 0.15 M NaCl and frozen in liquid nitrogen immediately. Note that during the centrifuge process, the loss of OD_600_ in the supernatant was measured for calibration. The protein samples could be stored in −80°C freezer before determination. 0.1 ml 3 M NaOH was added to protein samples for hydrolysis and then heated at 100°C for 5 min. Samples were then cooled in room temperature for 5 min. Subsequently, 0.1 ml 1.6% GuSO_4_ was added to each sample. The samples were further vortexed thoroughly and incubated for 5 min at room temperature. The tubes were further centrifuged at 12,000 rpm for 3 min and measured for OD_555_ with a Genesys50 UV‐Vis spectrophotometer (Thermo Sci). A series of bovine serum albumin (BSA) standard samples at the range of 0.4 to 2 mg/ml were also treated the same way to get a standard curve. Protein amounts were calculated according to the BSA standard curve.

### Measurement of the β‐galactosidase (LacZ) activity

Determination of β‐galactosidase activity was based on the classical Miller method as described.^68^ 0.5 ml of cell samples (OD_600_ of 0.3–0.5) were collected, rapidly frozen on liquid nitrogen, and stored at −80°C before β‐galactosidase assay. Three to four samples were collected during the exponential growth period. 0.5 ml Z‐buffer (containing 27 μl β‐mercaptoethanol per 10 ml buffer) and 0.5 ml cell sample were added to a reaction tube. Each sample was then supplemented with 25 μl 0.1% SDS and 50 μl chloroform. Tubes were vortexed for 10 s in the first round and 20 s in the second round for complete cell lysis and then incubated for 15 min at 37°C in water bath. 200 μl 2‐Nitrophenyl β‐d‐galactopyranoside (ONPG) (GlpBio) was then added, and the reaction mixtures were incubated until the development of yellow color. 0.5 ml 1 M Na_2_CO_3_ was then added to stop the reaction, and the reaction time was recorded. The reaction mixtures were then centrifuged (12,000 rpm, 10 min) and measured OD_420_.

### Proteomics

The proteomic study was based on 4D label‐free mass spectrometry approach following the protocol detailed,^5^ with key experimental steps being described again below. 40 ml *B. subtilis* cultures (OD_600_∼0.3) were harvested via centrifugation (4°C, 8500 rpm, 5 min) in pre‐chilled 50 ml tubes. Cell pellet was washed twice by PBS followed by dehydration using a CV600 speed vacuum concentrator (Beijing JM Technology Co., Ltd.). Cell pellets were stored at a −80°C freezer before further analysis. All proteomic sample preparation and mass spectrometry workflows were performed by JingJie PTM Bio lab (Hang Zhou) following the processes described^5^. Raw mass spectra were processed by the Maxquant software^69^ according to the SwissProt *B. subtilis* 168 databases, generating both label‐free quantification (LFQ) intensity values and intensity‐based absolute quantification (iBAQ) metrics. LFQ intensity gives the information of the relative abundance of each protein under different conditions, while iBAQ intensity is a proxy of the copy number of each protein^70^. To obtain the mass proteome fraction (absolute abundance) of each protein of *B. subtilis*, we used the values of iBAQ intensity to multiply the molecular weight of each protein to obtain the “iBAQ mass” value (Table S3). The “iBAQ mass” of each protein was further normalized by the sum of the “iBAQ mass” values of the whole proteome. The information of absolute abundance and gene locus‐tag were submitted to proteomaps website to obtain the resource allocation map of *B. subtilis* ^35^.

## AUTHOR CONTRIBUTIONS

**Haoyan Mu**: Data curation; formal analysis; investigation; methodology; resources; validation; writing—original draft. **Yiheng Wang**: Formal analysis; investigation; methodology; resources; validation; writing—original draft. **Xin Wang**: Investigation. **Xiongfeng Dai**: Conceptualization; data curation; formal analysis; funding acquisition; investigation; methodology; project administration; resources; supervision; validation; visualization; writing—original draft; writing—review and editing. **Manlu Zhu**: Conceptualization; data curation; formal analysis; funding acquisition; investigation; methodology; project administration; resources; supervision; validation; visualization; writing—original draft; writing—review and editing.

## ETHICS STATEMENT

This study did not involve any experiments on animals or humans.

## CONFLICT OF INTERESTS

The authors declare no conflict of interests.

## Supporting information

20250311 Supp information.

20250312‐Final Supplementary table of proteome.

## Data Availability

All data needed to evaluate the conclusions of this study are present in this paper. Proteome data have been deposited to the ProteomeXchange Consortium via the PRIDE partner repository. OG015LQ dataset: identifier PXD052459. XB04951LQ dataset: identifier PXD055309.
