# Supplementary material for: Condition‐dependent resource allocation strategy governed by CodY regulator in Bacillus subtilis
Source: mLife. 2025 Oct 22;4(5):539–50. doi: 10.1002/mlf2.70036 (PMC12575088; doi:10.1002/mlf2.70036)
Supplement: Supplementary file 1 — 20250311 Supp information. [file MLF2-4-539-s001.docx]

**Figure S1**. Typical exponential growth curves of wild type and *codY*-null strains under four different nutrient conditions. **(A)** LB rich medium; **(B)** glucose minimal medium; **(C)** mannose minimal medium; **(D)** arabinose minimal medium.

**Figure S2. Reproducibility of the proteome raw data.** Proteome data of both wild type strain and *codY-*null strain have been repeated twice, see supplementary tables for details. We analyzed the relative proteome mass of each individual proteins using the information of iBAQ intensity × MW (molecular weight) (see supplementary table 2). **(A)-(E)** Scatter plots between the data of two biological replicates for each condition confirm the high reproducibility of the proteome data. Panel A: two biological replicates of wild type 168 strain growing in LB medium; Panel B: two biological replicates of *codY*-null strain growing in LB medium; Panel C: two biological replicates of wild type 168 strain growing in glu cAA medium; Panel D: two biological replicates of *codY*-null strain growing in glu cAA medium; Panel E: two biological replicates of wild type 168 strain growing in glucose minimal medium. **(F-J)** Reproducibility of the proteome fractions of major proteome functional sectors for each condition. Panel F-H: wild type 168 strain growing in LB, glu cAA and glucose media, respectively. Panel I to J: *codY*-null strains growing in LB and glucose cAA media, respectively.

**Figure S3.** The relative level of individual ribosomal proteins and translation factors in wild type and *codY*-null strain. Data are based on LFQ intensity. (A) 50S ribosomal proteins for cells growing in LB medium; (B) 30S ribosome proteins and translation factors for cells growing in LB medium; (C) 50S ribosomal proteins for cells growing in glu+cAA medium; (D) 30S ribosome proteins and translation factors for cells growing in glu+cAA medium.

**Figure S4.** The mass fractions of each non-BCAA biosynthetic pathways in wild type and its *codY*-null strains growing under LB broth or glu cAA medium. (A) Data of LB broth; (B) Data of glu cAA medium.

**Figure S5.** The relative level of individual amino acid biosynthetic proteins in wild type and *codY*-null strain. Data are based on LFQ intensity. (A)-(B) Data for cells growing in LB medium; (C)-(D) Data for cells growing in glu+cAA medium.

**Figure S6.** The relative level of individual proteins belonging to CodY regulon in wild type strain and *codY*-null strain. Data are based on LFQ intensity. (A) Motility proteins belonging to CodY regulon; (B) Proteins involved in oligopeptide transport and secondary metabolite biosynthesis belonging to CodY regulon; (C) Protein involved in alternative nutrient utilization for cells growing in LB medium. (D) Protein involved in alternative nutrient utilization for cells growing in glu cAA medium.

**Figure S7.** The proteome fractions of Ribosomal proteins and AA biosynthetic proteins of wild type strain and *codY*-null strain under different conditions. In addition to Figure 4a, here we show more details of ribosomal proteins and AA biosynthetic proteins including 30S ribosome subunits, 50S ribosome subunits, translation factors and various specific categories of amino acid biosynthetic proteins. Note that the data of non-BCAA biosynthesis shown in panel C are the sum of individual components shown from panel D to N.
